# Paper-based geriatric interprofessional education for enhancing students’ interest in treating older people

**DOI:** 10.12688/mep.19773.1

**Published:** 2024-04-24

**Authors:** Carolyn Joyce Teuwen, Karlijn Vorstermans, Rashmi A. Kusurkar, Hermien Schreurs, Hester E.M. Daelmans, Saskia M. Peerdeman

**Affiliations:** 1 Northwest Academy, Northwest Clinics Alkmaar, P.O. box 501, 1800AM, Alkmaar, North Holland, The Netherlands; 2 Research in Education, Amsterdam UMC location Vrije Universiteit Amsterdam, De Boelelaan 1118, Amsterdam, North Holland, The Netherlands; 3 Department of Geriatrics, OLVG hospital Amsterdam, Amsterdam, North Holland, The Netherlands; 4 LEARN! Research Institute for Learning and Education, Faculty of Psychology and Education, Vrije Universiteit Amsterdam, Amsterdam, North Holland, The Netherlands; 5 Amsterdam Public Health, Quality of Care, Amsterdam, North Holland, The Netherlands; 6 Department of Surgery, Northwest Clinics Alkmaar, Alkmaar, North Holland, The Netherlands; 7 Faculty of Medicine, Vrije Universiteit Amsterdam, Amsterdam, North Holland, The Netherlands; 8 Teaching & Learning Centre (TLC) FdG - UvA, Amsterdam UMC location AMC, Amsterdam, North Holland, The Netherlands; 9 Department of Neurosurgery, University of Amsterdam, Amsterdam UMC location Vrije Universiteit Amsterdam, Amsterdam, North Holland, The Netherlands

**Keywords:** Interprofessional education, attitudes, older patients, nursing students, medical students, controlled intervention study

## Abstract

Interprofessional education is one of the interventions used to increase health care students’ motivation for working with older patients. Previous research about such interventions has been conducted without the use of control groups and has given inconclusive results. The objective of the present curricular resource was: Does geriatric paper-based interprofessional education influence students’ interest in treating older people? During a one-year period, undergraduate fourth-year medical and third-year nursing students wrote four health care plans for four different paper-based older patient cases. In the intervention group students were paired up in interprofessional couples. In the control group students made the assignment alone. Interest for working with older patients was measured on a 5-point Likert scale before and one year after the intervention. In both groups, no significant change was found. Before-interest score of the interprofessional group was relatively high (3.8) so the non-significant results may be due to a ceiling effect. Nursing students’ interest in treating older people at the start of the research was higher than medical students’ interest.

## Introduction

Healthcare workers specialized in treating older patients are urgently needed, but there is a lack of interest among students for doing this ^1, 2^. WHO suggests interprofessional education (IPE) and collaborative practice as the most promising solutions to overcome this shortage ^3^. Since a variety of disciplines are involved in geriatric care, IPE is well-matched with geriatric care education. The combination of IPE and geriatric care education has been studied together in different settings, with different aims ^4– 8^. Only a few studies have investigated if IPE in geriatric care improves students’ motivation for working with older patients. These studies have been conducted without a control group and have given inconclusive results ^9– 13^. Whether IPE improves students’ motivation to work with older patients remains unanswered. Therefore, the research question for this study was:

Does geriatric problem-based interprofessional education influence medical and nursing students’ interest in treating older patients?

## Methods

### Participants

Two groups of students were included in this study: (1) Undergraduate third-year nursing students, in a four-year educational program (classroom education alternates with clinical practice). They had finished a theoretical geriatrics course. Three groups of nursing students (maximum 24 students per group) were asked to participate in this study. The nursing students were individually randomly assigned to the intervention or control group; (2) Undergraduate fourth-year medical students who were starting their master’s program, consisting of different clerkships alternated with a few weeks of classroom based teaching, including a clerkship care for older people in a nursing home. Every six weeks a group of maximum nine students started their clerkships at our educational facility. All groups that started between March 2018 and March 2019 were asked to participate in the study and were assigned as a group to the intervention or control group based on their schedule and if that could match with one of the nursing groups.

Students had no prior interprofessional education experience.

### Setting and assignment

At the Northwest Clinics in Alkmaar, the Netherlands, the students were asked to draw up health care plans for four paper-based geriatric patient cases, over a one-year period. The four cases, with an increasing level of difficulty and typical geriatric care problems, were constructed in collaboration with several geriatric experts ^14^. A patient case included information about the patient’s background, medication, description of the current health problem, social and functional status and some results from a physical examination.

Students were asked to draw up a health care plan ‘like they would in clinical practice’. They were allowed the use of books or guidelines, but were not allowed to discuss with others (except their assigned partner if they were in the intervention group). In the instructions the students were suggested to think of diagnostics, medication, consultation of different specialists, nursing interventions and ‘to-do’ tasks before discharge.

**Figure 1.**
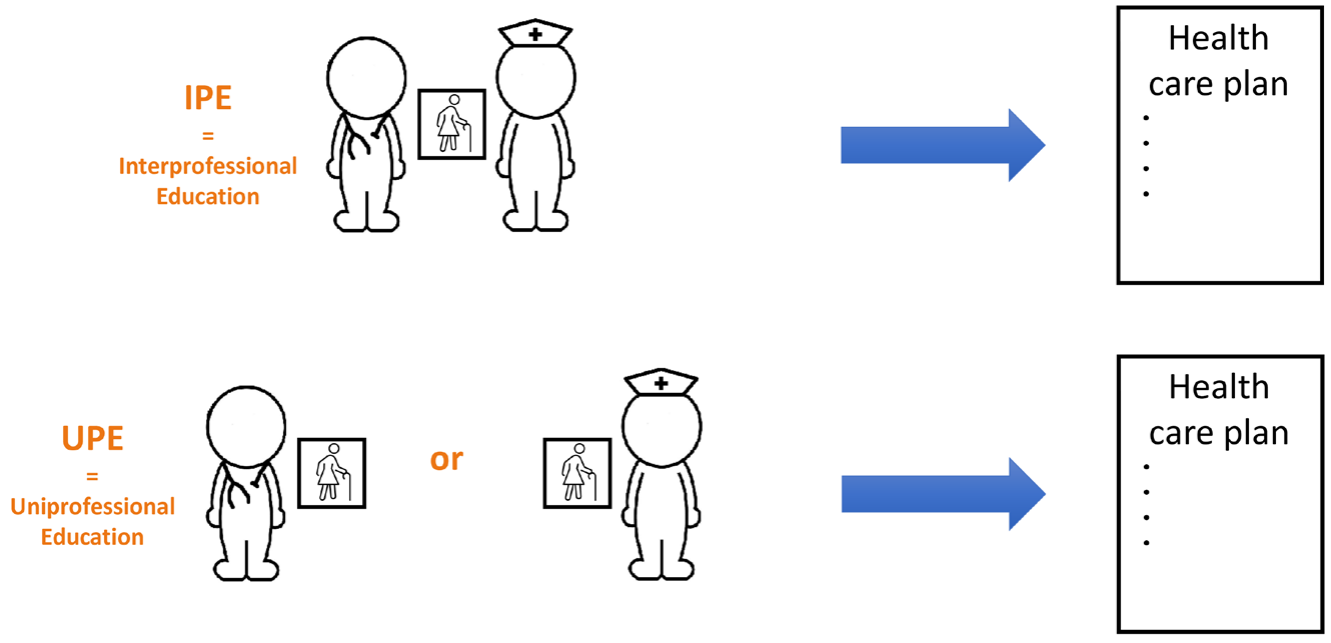
Visualisation of the intervention and control group.

In the control group, students wrote the health care plans on their own (uniprofessional education group, i.e., UPE-group). In the intervention group, the health care plans were written by randomly paired medical and nursing students (interprofessional education group, i.e., IPE-group) ( Figure 1). In each session different pairs were assembled to create diversity among the collaboration partners. The intervention group was placed in a different (class)room, separate from the control group. The scheduled time for the assignment was 45 minutes. A researcher or research assistant supervised the students for identifying (non-)collaboration and was available to answer procedural questions.

**Figure 2.**
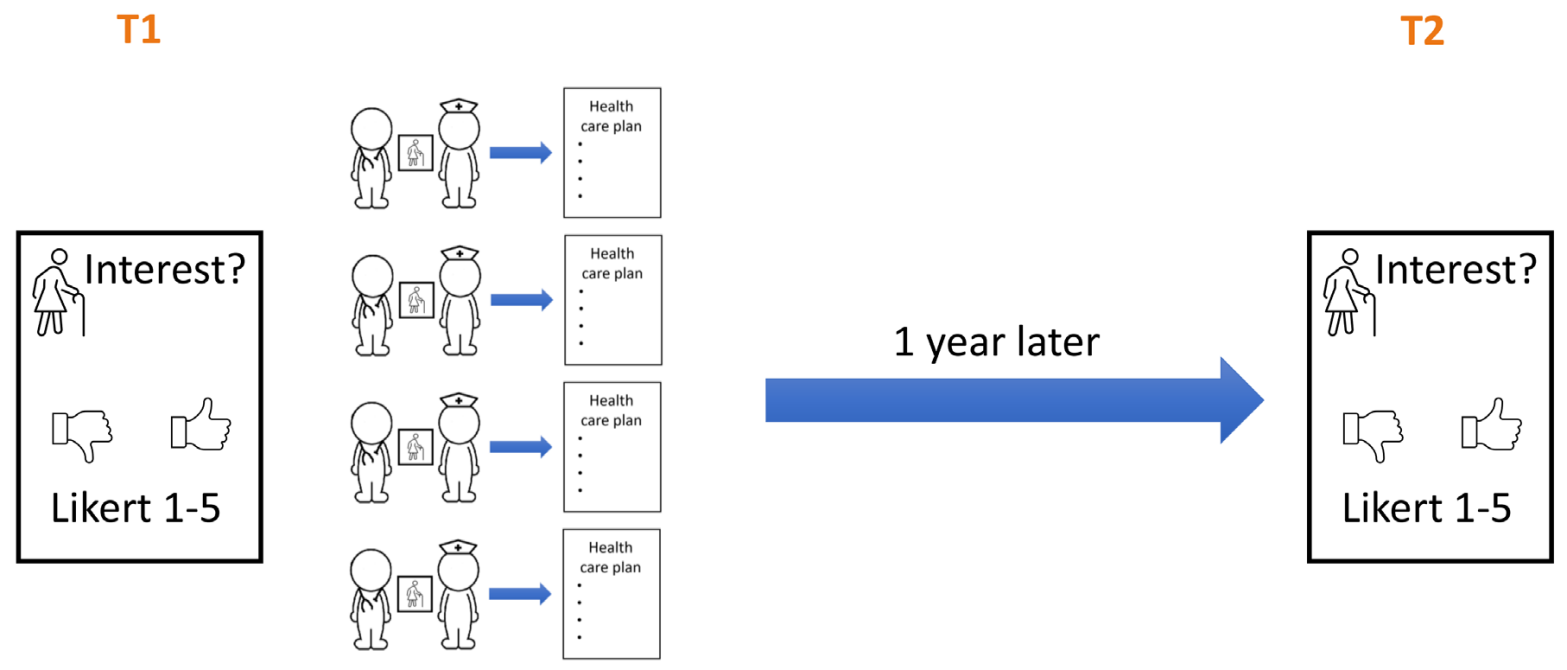
Timing of the before (T1) and after (T2) questionnaires.

Before (T1) and one year after the intervention (T2) all students reported their interest in working with children, adults and older patients on a 5-point Likert scale ( Figure 2). At T1 the questionnaire was handed out in person. At T2 the students received the questionnaire by email.

### Data analysis

SPSS version 28.0.0.0 was used for analysis. Before and after interest scores were compared using Paired t-tests.

### Ethical approval

The study was submitted to the local scientific board of Northwest Clinics, the Netherlands, which considers all study proposals within the institution. Since the study does not fall within the scope of the Dutch Medical Research Involving Human Subjects Act (section 1.b WMO, 26th February 1998), the local scientific board of Northwest Clinics approved the study and waived the need for approval by an Ethics Committee/Institutional Review Board. All participants received a research information letter and signed an informed consent form. Participation was voluntary. Written informed consent was obtained from all participants for using their anonymous data in our study. All methods were performed according to the relevant guidelines and regulations.

## Results

One hundred and twenty-nine students filled out the T1 questionnaire, and sixty-two (48%) filled out the T2 questionnaire. Two completed questionnaires lacked a research identification number, so sixty students were included in the final analysis.

In the total group (n=129) there were no differences between the UPE- and IPE-groups. Within the group included in the analysis (n=60), we found differences between the UPE- and IPE-groups ( Table 1).

**Table 1.** Total group characteristics and characteristics of the group included in the analysis.

	Total group, n=129			*Included in analysis, n=60*			Test
	UPE	IPE	p-value	*UPE*	*IPE*	*p-value*	
	n=68	n=61		*n=31*	*n=29*		
**Nursing students n(%)**	29(43%)	30(49%)	0.483	*12(39%)*	*19(66%)*	0.044 *	Fisher’s exact
**Female n(%)**	57(84%)	44(72%)	0.135	*29(94%)*	*21(72%)*	0.039 *	Fisher’s exact
**Age in years (mean ±SD)**	21.8 ±2.8	21 ±2.6	0.091	*22 ±2.9*	*20.5 ±2.7*	0.044 *	T-test
**Prior experience older adults n(%)**	42(62%)	34(58%)	0.591	*19(61%)*	*20(69%)*	*0.595*	Fisher’s exact
**Interest score T1 (1-5) (mean ±SD)**	3.1 ±1.1	3.4 ±1.1	0.140	*3.1 ±1.2*	*3.8 ±0.9*	0.013 *	T-test

UPE=uniprofessional education, IPE=interprofessional education *p<0.05

In both groups the interest in treating older patients did not change significantly after the intervention. The only significant result was a decrease in interest in treating children in the UPE-group ( Table 2).

**Table 2.** Mean interest in treating different patient groups at T1 and T2 (paired t-test).

		UPE-group	IPE-group
		n=31	n=29
***Older adults***	*T1*	3.1 ±1.2	3.8 ±0.9
	*T2*	3.5 ±1.0	3.8 ±1.0
	p-value	0.076	0.873
		N=16 **	N=24 **
***Adults***	*T1*	4.4 ±0.6	4.3 ±0.9
	*T2*	4.7 ±0.5	4.6 ±0.6
	p-value	0.055	0.119
		N=17 **	N=24 **
***Children***	*T1*	4.1 ±1.1	3.8 ±1.0
	*T2*	3.1 ±1.2	3.4 ±1.2
	p-value	0.001 *	0.067

The p-value of each paired t-test describes whether the difference between the scores at T1 and T2 within each group is significantly different. UPE=uniprofessional education, IPE=interprofessional education, T1=before the intervention, T2=one year after the intervention *p<0.05 **Not all students filled out the section about interest in treating adults and children, so the response rates between all questions differ.

## Discussion

In this study we found no significant change in interest in treating older patients before and after the intervention, in both the UPE- and IPE-groups. This lack of change could have been caused by a couple of factors.

First, the UPE- and IPE-group were not comparable anymore at T2. Most important difference between the two groups was the proportion of nursing students (39% in the UPE-group versus 66% in the IPE-group, p=0.044). We conducted further analyses to investigate the correlations. The nursing students were significantly younger than the medical students (19.4 versus 23.1 years, p<0.001), which caused the significant age difference between the groups. Also, the interest score at T1 differed between the nursing and medical students (3.8 versus 3.0, p=0.012). Consequently, the bigger proportion of nursing students was probably the reason of the higher interest score at T1 in the IPE-group. The differences between nursing and medical students must be taken into account in future research about interest in geriatrics.

Second, the lack of change in interest in especially the IPE-group could have been caused by a ceiling effect. Hughes *et al.* ^15^ also scored fourth year students’ attitudes towards older patients after a geriatric educational intervention and found similar scores (3.86). Maybe higher mean interest scores are impossible to find, not even with the most effective interventions.

In our study, the only significant result was the decline in interest in treating children in the uniprofessional group. Earlier research has also found such trends in interest: downwards for children, upwards for older people, among both medical and nursing students ^16– 19^. These trends are without interventions and happen just as a result of progression in students’ education. This emphasizes the importance of the usage of a control group, because students’ interests and preferences change over time.

Besides interprofessional education, several other types of interventions have been studied aiming to increase students’ interest in older patients, such as ‘older adult mentor programs’, geriatric courses and home visits ^20– 22^. Tullo *et al.* ^21^ describe that longer rather than shorter interventions, and interventions that involve healthy older adults instead of older patients in a clinical environment, are more likely to improve students’ attitudes towards older adults. Meiboom *et al.* ^23^ emphasize a more rigorous and bigger curricular change instead of low impact discrete interventions to motivate students for the medical care for older patients.

This study is subject to some limitations. First, due to a low response rate at T2, the study has a small sample size. Second, although the intervention was spread out over a longer period, it was relatively small (4 sessions). Third, we measured interest after one year, so it is possible that the intervention did influence students’ interest directly after, but this effect was not sustained. Maybe an intervention with more or longer sessions is necessary to make a change sustainable.

## Ethics and consent

The study was submitted to the local scientific board of Northwest Clinics, the Netherlands, which considers all study proposals within the institution. Since the study does not fall within the scope of the Dutch Medical Research Involving Human Subjects Act (section 1.b WMO, 26th February 1998), the local scientific board of Northwest Clinics approved the study and waived the need for approval by an Ethics Committee/Institutional Review Board. All participants received a research information letter and signed an informed consent form. Participation was voluntary. Written informed consent was obtained from all participants for using their anonymous data in our study. All methods were performed according to the relevant guidelines and regulations.

## Data Availability

The privacy officers of Amsterdam UMC advised not to share the data due to ethical considerations: we didn’t asked students their permission at the start of the study, and we are not able to do it retroactively. At the start of our study, all students signed an informed consent form, but we didn’t ask the students their permission to share the data with others. At that time open data sharing wasn’t common. All students are now out of reach, so asking their permission afterwards is not an option. We anonymized the file, but because it is a small sample size, and we included two different groups of students (medical and nursing), students (or others) might be able to identify themselves. No intermediary data can be de-identified without compromising anonymity. Thus, there are no conditions under which access will be granted.
