# Supplementary material for: Geriatric interprofessional education for enhancing students’ interest in treating older people
Source: MedEdPublish (2016). 2024 Sep 12;14:24. Originally published 2024 Apr 24. [Version 2] doi: 10.12688/mep.19773.2 (PMC11443234; doi:10.12688/mep.19773.2)
Supplement: Peer review history

# Reviewer report: Reviewer response for version 2

**DOI:** 10.21956/mep.22037.r39477

**Published:** 2025-01-16

**Authors:** Andrea Wershof Schwartz

**Affiliations:** 1 VA Boston Healthcare System, and Brigham and Women’s Hospital, Boston, USA

Thank you for this revision which has greatly improved the paper. The methods are very well done and the discussion and limitations section is particularly strong and points to the need for further investigation on the impact of this type of educational intervention. I have no further comments and appreciate the opportunity to review this excellent work.

Have any limitations of the research been acknowledged?

Yes

Is the study design appropriate and does the work have academic merit?

Yes

Is the work clearly and accurately presented and does it cite the current literature?

Yes

If applicable, is the statistical analysis and its interpretation appropriate?

Yes

Are all the source data underlying the results available to ensure full reproducibility?

Yes

Are the conclusions drawn adequately supported by the results?

Yes

Are sufficient details of methods and analysis provided to allow replication by others?

Yes

Reviewer Expertise:

geriatrics, medical education

I confirm that I have read this submission and believe that I have an appropriate level of expertise to confirm that it is of an acceptable scientific standard.

# Reviewer report: Reviewer response for version 2

**DOI:** 10.21956/mep.22037.r40382

**Published:** 2025-01-08

**Authors:** W.P. Achterberg, Annemarie Moll-jongerius

**Affiliations:** 1 Leiden University Medical Center, Leiden, The Netherlands

The authors describe an intervention in medical education to investigate the influence of interprofessional education on the interest of medical students and nursing students to work with older patients. This intervention includes working groups in which medical students and nursing students draw up health care plans concerning four geriatric patient cases over a period of one year. To investigate the influence of interprofessional education and collaboration they use an intervention group and control group. The authors explain that this method hasn’t been used before to evaluate this influence of IPE.

 First of all, I want to complement and thank the authors for this work. Research on geriatric medical education is still scarce, the care for older persons is not the first choice for medical students and interprofessional collaboration is an important development in older persons’ healthcare. After reading the article I have a few questions, especially on the content of the introduction and the considerations in the discussion.

**Introduction:**

 in the introduction is mentioned that ‘WHO suggests interprofessional education…to overcome this shortage’ and ‘Only a few studies…for working with older patients. Can you explain little more about these two conclusions. Why does IPE improve students’ motivation for working with older persons? 

**Study design:**

 Doing a trial with an intervention and control group is very important if one wants to gain more objective knowledge about the effect of the specific intervention. Some very important elements that should be acknowledged in the design are finding a good outcome measure, with enough room for improvement. Related to that, is that one should try to do a power analysis. Also, one should try to make the difference between intervention and control only to appear in the specific intervention, in this study the interprofessional way of cooperation. However, as I understood, there now also was the difference of working alone, or in a pair.

 To better understand the influence of interprofessional education on the interest in treating older patients I would suggest using a mixed method study. The lack of quantitative precision could have been compensated by more in depth qualitative information.

 In the discussion several assumptions are made. For example, ‘Perhaps, even with the most successful…. are unachievable’ or ‘Perhaps, even with…. led to different results. With a qualitative component you can better explore why some results arise. I suggest that you reflect on these methodological issues in your limitations.

**Results:**

 Can you include a table with the group characteristics of medical students and nursing students? You mentioned some of these characteristics in your discussion.

**Discussion:**

 No significant change in interest is measured and some possible reasons of this result are described in the discussion. I would like to read more about the literature that is related to your study topic. Therefore, my first question is ‘what is known in the literature about the difference between nursing students and medical students concerning the interest for older persons health care?’ And is there a relation with interprofessional collaboration? Furthermore, can you explain the ceiling effect more deeply and relate this also to your topic IPE, and suggest a better quantitive outcome measure for future studies? My last question is ‘can you further explain which interventions are mentioned in the article of Tullo *et al*. and also relate this to your topic IPE?

Have any limitations of the research been acknowledged?

Partly

Is the study design appropriate and does the work have academic merit?

Partly

Is the work clearly and accurately presented and does it cite the current literature?

Partly

If applicable, is the statistical analysis and its interpretation appropriate?

Partly

Are all the source data underlying the results available to ensure full reproducibility?

Yes

Are the conclusions drawn adequately supported by the results?

Partly

Are sufficient details of methods and analysis provided to allow replication by others?

Partly

Reviewer Expertise:

NA

We confirm that we have read this submission and believe that we have an appropriate level of expertise to confirm that it is of an acceptable scientific standard, however we have significant reservations, as outlined above.

# Reviewer report: Reviewer response for version 2

**DOI:** 10.21956/mep.22037.r39214

**Published:** 2024-12-12

**Authors:** Andrea Justine Landi

**Affiliations:** 1 University School of Chicago School of Medicine, Chicago, Illinois, USA

I want to first thank the authors for their work in interprofessional education, as well as focusing on the care of older adults. The authors evaluated an educational program that compared students’ interest in older adults after interprofessional vs uniprofessional case-based learning. The authors conclude that the potential reasons for a lack of difference between pre- and post-intervention interest in older adults are:

1. differences in the proportion of nursing compared to medical students,
2. a ceiling effect, and
3. the nature of the intervention.

This is an important area of geriatrics education research that is much needed.

 General comments: 1) recommend reviewing the article to ensure that terms are being used consistently, 2) and reviewing the article for grammatical errors (i.e., discussion section – Paragraph that starts with “Third, it is possible…is caused by the nature **of** the intervention).

**Introduction:**

- For the question at the end – is it problem-based or would it be better described as “case-based”?

**Methods:**

- **Participants:**
  - Are both nursing and medical school programs four years? If so, what was the rationale for choosing third year nursing students and fourth year medical students?
  - UPE is used before its meaning is clarified later one.
  - Consider adding more details regarding your educational facility and the types of learning experiences for both nursing and medical students (i.e., inpatient, outpatient primary care, nursing home).
- **Setting and Assignment:**
  - Recommend clarity regarding the case-based learning intervention, such as how were the four cases distributed through the year? Quarterly? How did this take place if their rotation on the clerkship was only 6 weeks?
  - A main concern of mine is the single question used “How interesting do you find these patient groups?” I agree with other reviewers in learning more about the origin of this survey question and the testing of this survey instrument, as well as considering having more than one question to assist in answer your research question. My other concern is the wording and asking students if they find older adults interesting. I understand the question you are trying to ask, but I worry it might not be able to answer the question you are asking. I do not have a solution readily available, but I do think there are ways to identify what makes the work challenging/discouraging or rewarding. Or ways to identify their knowledge gaps that influence their sense of satisfaction or reward with caring for older adults that working with other disciplines (interprofessional collaboration) could help with. I do not think the wording of this question takes away from the impact of this research rather I think it highlights the difficulty in this research. I believe the authors’ work does add to the research of how to evaluate this concept in learners.
- **Results:**
  - The section, including Figure 1, would provide more details regarding participants. The Figures (1 and 2) are easy to understand but do not add much in terms of my understanding of the intervention.
  - It might be worth noting the fact that no change in interest was seen in any of the IPE groups. (older adults, adults, or children). That might be more applicable to this paper than commenting on the decrease in interest in treating children in the UPE group.
- **Discussion:**
  - I wonder if the authors can provide additional thoughts as to why the larger proportional of nursing students would be a reason for a higher interest score at T1 in the IPE group? Why would this explain the higher score in general? Also – why would it just be in T1 and not T2? And why would it be in IPE and not UPE?
  - Also, for the analysis of nursing and medical students – the authors might consider describing this in a table as well.
  - I recommend considering the following comments to further strengthen the conclusions:
     
    - Regarding the conclusion that the ceiling effect could explain the lack of change - my concern is that the students were only ranking a 3 for their interest which I assume was a “neutral” response. This would be discouraging if this was pre-intervention was the highest possible score expected from students. I question this as a possible conclusion.
    - Does social desirability bias play a role?
    - Does non-response bias play a role?
    - Also, it might be worth considering that the lack of change in interest after one year of case-based IPE learning could be because of aspects not related to this intervention, like other clinical experiences. Or even the fact that interprofessional collaboration is a skill that is developed over time and the students may be novices in this area and therefore impacting the overall impact of the intervention.

Have any limitations of the research been acknowledged?

Partly

Is the study design appropriate and does the work have academic merit?

Partly

Is the work clearly and accurately presented and does it cite the current literature?

Yes

If applicable, is the statistical analysis and its interpretation appropriate?

Partly

Are all the source data underlying the results available to ensure full reproducibility?

Partly

Are the conclusions drawn adequately supported by the results?

Partly

Are sufficient details of methods and analysis provided to allow replication by others?

Partly

Reviewer Expertise:

I am a clinician educator in geriatrics. A limitation to my review is in the area of statistical analysis.

I confirm that I have read this submission and believe that I have an appropriate level of expertise to confirm that it is of an acceptable scientific standard, however I have significant reservations, as outlined above.

# Reviewer report: Reviewer response for version 2

**DOI:** 10.21956/mep.22037.r38789

**Published:** 2024-11-01

**Authors:** Rilani Riskiyana

**Affiliations:** 1 Department of Medical Education and Bioethics, Gadjah Mada University, Yogyakarta, Special Region of Yogyakarta, Indonesia

Appreciation to the authors for addressing the suggestions into the new version.

 One more question and suggestion regarding the quality of the instrument.

- Did you do a pilot study? If not, then you need to address it in limitation.

Have any limitations of the research been acknowledged?

Partly

Is the study design appropriate and does the work have academic merit?

Partly

Is the work clearly and accurately presented and does it cite the current literature?

Yes

If applicable, is the statistical analysis and its interpretation appropriate?

Partly

Are all the source data underlying the results available to ensure full reproducibility?

Partly

Are the conclusions drawn adequately supported by the results?

Partly

Are sufficient details of methods and analysis provided to allow replication by others?

No

Reviewer Expertise:

interprofessional education

I confirm that I have read this submission and believe that I have an appropriate level of expertise to confirm that it is of an acceptable scientific standard, however I have significant reservations, as outlined above.

# Reviewer report: Reviewer response for version 2

**DOI:** 10.21956/mep.22037.r39091

**Published:** 2024-10-02

**Authors:** C.J.P.W. Keijsers Keijsers

**Affiliations:** 1 Jeroen Bosch Hospital, ME ‘s-Hertogenbosch, The Netherlands

First, I would like to complement the authors with this work which is highly relevant. It is well written and interesting. The figures are very beautiful and clear. However, this was the first time that I read the paper, and some point could be clarified.

1. Some abbreviations are introduced without spelling them out first (UPE). Probably, this is the result of previous revisions. Please check the full paper "like you've never read it before" on readability. 
2. The result section could be improved. I do think the results are accurate, but the readability could be improved. The tables aren't clear enough without further explanation in text while it should be clear on its own. 
  To specify:
  a) why are so many characteristics given of those not included in the analyses. Isn't that confusing?
  b) please specify that one is the before and the other is the after measure.
  c) Please verify what is compared to what. Additional question: was a repeated measure ANOVA considered since you have two groups before and two groups after.

The discussion section is good, no additional advises. 

 To take above together, it is a good paper, but the readability needs small point of improvement.

 Perhaps one additional citation could be used.

Have any limitations of the research been acknowledged?

Yes

Is the study design appropriate and does the work have academic merit?

Yes

Is the work clearly and accurately presented and does it cite the current literature?

Yes

If applicable, is the statistical analysis and its interpretation appropriate?

Partly

Are all the source data underlying the results available to ensure full reproducibility?

Yes

Are the conclusions drawn adequately supported by the results?

Partly

Are sufficient details of methods and analysis provided to allow replication by others?

Yes

Reviewer Expertise:

Geriatric medicine

I confirm that I have read this submission and believe that I have an appropriate level of expertise to confirm that it is of an acceptable scientific standard, however I have significant reservations, as outlined above.

## References

[1] : Interprofessional education in geriatric medicine. *European Geriatric Medicine* .2016;7(4) : 10.1016/j.eurger.2016.01.011 306-314 10.1016/j.eurger.2016.01.011

# Reviewer report: Reviewer response for version 2

**DOI:** 10.21956/mep.22037.r38831

**Published:** 2024-09-30

**Authors:** Christina Parker

**Affiliations:** 1 Queensland University of Technology, Brisbane, Queensland, Australia

Noting this is the first time that I have read this article, however I have read previous reviewer's comments and also author replies. This is an important area of education and needs more evidence and incorporation into health programs. 

 The article would benefit from some further edits.

**Presentation:**

- While this is well written there are some areas of inconsistency ie older patient, geriatric patient, older people – need to be consistent and I would suggest they are people not patients. 
- There needs to be a definition of IPE and the acronym IPE is not consistently used throughout.
- For an international audience you need to explain terms such as clerkship.  That is not a term that I am familiar with.
- I note the title has been changed but paper based is still intermittently used throughout the paper.  I’d suggest this is a method and should be explained there and is not needed elsewhere.

**Study Design:**

- Overview of student numbers is still unclear; table needs to be explained.  I’m still not clear on how many students were invited to participate.

**Methods and analysis:**

- Why one year follow up? 
- Were they still students at that point? 
- If not, what impact could this have had? 
- Why only one question – wouldn’t asking other questions potentially about participant characteristics have added to the analysis?

**Conclusion:**

- The discussion brings up new information which should not occur in a discussion i.e. bigger proportion of nursing students – where is this mentioned in results?  Why would this be a reason for higher interest scores? 
- You mention a score of 3.86 in another study but no indication of whether you are comparing the same scale.

Have any limitations of the research been acknowledged?

Partly

Is the study design appropriate and does the work have academic merit?

Partly

Is the work clearly and accurately presented and does it cite the current literature?

Partly

If applicable, is the statistical analysis and its interpretation appropriate?

Yes

Are all the source data underlying the results available to ensure full reproducibility?

Yes

Are the conclusions drawn adequately supported by the results?

Partly

Are sufficient details of methods and analysis provided to allow replication by others?

Partly

Reviewer Expertise:

NA

I confirm that I have read this submission and believe that I have an appropriate level of expertise to confirm that it is of an acceptable scientific standard, however I have significant reservations, as outlined above.

# Reviewer report: Reviewer response for version 1

**DOI:** 10.21956/mep.21187.r37137

**Published:** 2024-06-18

**Authors:** Rilani Riskiyana

**Affiliations:** 1 Department of Medical Education and Bioethics, Gadjah Mada University, Yogyakarta, Special Region of Yogyakarta, Indonesia

The paper posed potential improvement for IPE benefit on geriatric course.

 Some suggestions for further improvement:

 1. What is paper-based IPE and what is the theoretical pedagogy for this? Need to elaborate on the background.

 2. Methods Section, Participants subsection: need to report how the nursing and medical students were mixed and divided into the IPE and UPE groups. How many of them are in IPE and UPE groups? Were they divided into smaller groups? if so how many team members are there in each small group?

 3. Figure 1 needs to provide information in detail, e.g. number of participants in each group, and the detailed intervention.

 4. Data analysis, is the data distribution normal? Were the assumptions for parametric testing met? Need to provide these information in the report.

 5. How valid was the instrument being used? Was it self-developed or using an established instrument? What was the internal consistency?

 6. When the sample size is unequal (as stated in the third sentence in the Discussion section), the authors need to test the variance, report the result, and then use the most appropriate statistical testing to compare means in T2 between UPE and IPE groups when the sample size is unequal and heterogeneity between groups is identified.

 7. May need to revise the discussion accordingly.

 8. The authors state "We conducted further analysis to investigate the correlations.". What variables being examined for their correlations? What testing was used? Need to report in the data analysis section. The sentence was then followed by a report on the age comparison between nursing and medical students, but the report was on the p-value instead of the r-statistic (correlation). To investigate correlation, the authors need to have at least two different variables being examined. Testing the age variable (only one variable) and comparing the difference between two groups of participants is not a correlation.

Have any limitations of the research been acknowledged?

Partly

Is the study design appropriate and does the work have academic merit?

Partly

Is the work clearly and accurately presented and does it cite the current literature?

Yes

If applicable, is the statistical analysis and its interpretation appropriate?

Partly

Are all the source data underlying the results available to ensure full reproducibility?

Partly

Are the conclusions drawn adequately supported by the results?

Partly

Are sufficient details of methods and analysis provided to allow replication by others?

No

Reviewer Expertise:

interprofessional education

I confirm that I have read this submission and believe that I have an appropriate level of expertise to confirm that it is of an acceptable scientific standard, however I have significant reservations, as outlined above.

# Reviewer report: Reviewer response for version 1

**DOI:** 10.21956/mep.21187.r37136

**Published:** 2024-06-17

**Authors:** Anne W

**Affiliations:** 1 Chapman University, Orange, California, USA

The abstract gives a clear summary of the article. Over one year, fourth-year medical and third-year nursing students were compared in intervention (MD plus RN) and control (solo) groups to write four health care plans for four different geriatric paper cases. Interest for working with older patients was measured by self-report and no significant change was found between groups. Authors theorize that this may be due to a ceiling effect, particularly with RN students whose baseline interest in geriatrics was higher than medical student interest.

 Current, known research was cited and appropriately applied to the research question: “Does geriatric problem-based interprofessional education influence medical and nursing students’ interest in treating older patients?” The design was a controlled experimental study, which was appropriate to the question. Methods are easily reproducible, and statistical analysis and interpretation was appropriate and correct. Limitations were identified and conclusions were valid.

 The writing was appropriate except for minor issues with casual language use in the Discussion section, e.g. “a couple of factors” and “Maybe higher means scores are harder to find.” It is interesting that the only significant finding was a decline in interest for treating pediatric patients. Is this proportional to any increase, even if statistically insignificant, in treating geriatric patients (in other words, not a true decrease, simply a proportional issue?). Worth commenting on in the discussion section.

 Lastly, I recommend clarity regarding whether IPE itself makes no statistically significant difference, vs. IPE with paper cases instead of real patients makes no statistically significant difference. In other words, is it the IPE or the paper? My hypothesis is that if students were working with 4 standardized or real patients over the same time period with the same exercise, there may be more of a difference. This could be mentioned as an opportunity for further research if there are no current studies of this nature.

 As an IPE/patient outcomes researcher, I thank you for the opportunity to review this submission.

Have any limitations of the research been acknowledged?

Yes

Is the study design appropriate and does the work have academic merit?

Yes

Is the work clearly and accurately presented and does it cite the current literature?

Yes

If applicable, is the statistical analysis and its interpretation appropriate?

Yes

Are all the source data underlying the results available to ensure full reproducibility?

Yes

Are the conclusions drawn adequately supported by the results?

Yes

Are sufficient details of methods and analysis provided to allow replication by others?

Yes

Reviewer Expertise:

IPE: student attitudes, student abilities, and patient outcomes.

I confirm that I have read this submission and believe that I have an appropriate level of expertise to confirm that it is of an acceptable scientific standard.
